# Two dominant genes in barley (*Hordeum vulgare* L.) complementarily encode perfect resistance to *Japanese soil-borne wheat mosaic virus*

**DOI:** 10.1270/jsbbs.22046

**Published:** 2022-12-13

**Authors:** Kaori Okada, Tsuyoshi Tanaka, Shuichi Fukuoka, Youko Oono, Kohei Mishina, Tetsuo Oikawa, Kazuhiro Sato, Tsuneo Kato, Takao Komatsuda, Kiyoshi Namai

**Affiliations:** 1 Tochigi Prefectural Agricultural Experiment Station, 1080 Kawaraya-cho, Utsunomiya, Tochigi 320-0002, Japan; 2 Research Center for Advanced Analysis, National Agriculture and Food Research Organization (NARO), Kan-non-dai, Tsukuba, Ibaraki 305-8518, Japan; 3 Institute of Crop Science, National Agriculture and Food Research Organization (NARO), Kan-non-dai, Tsukuba, Ibaraki 305-8518, Japan; 4 Institute of Plant Science and Resources, Okayama University, 2-20-1 Chuo, Kurashiki, Okayama 710-0046, Japan; 5 Graduate School of Horticulture, Chiba University, Matsudo, Chiba 271-8510, Japan; 6 Crop Research Institute, Shandong Academy of Agricultural Sciences (SAAS), 202 Gongyebei Road, Licheng District, Ji’nan, 250100 Shandong, China

**Keywords:** Soil-borne disease, *Furovirus*, *Polymyxa graminis*, QTL and genetic mapping, dominance

## Abstract

*Japanese soil-borne wheat mosaic virus* (*Furovirus*) is a damaging pathogen of wheat and barley. This virus can survive in the soil for several decades, so the deployment of resistant cultivars represents the only practical control measure. Here, a genetic analysis has identified two regions of the barley genome—one on chromosome 2H and the other on chromosome 3H—as harboring gene(s) encoding resistance to this virus. The joint presence of both loci, termed *Jmv1* and *Jmv2*, made the plants essentially immune, with resistance being dominant over susceptibility at each locus. Phylogenetic analysis showed that the virus is not closely related to the type *Furovirus* species *Soil-borne wheat mosaic virus*. There was a difference between the RNA1- and RNA2-based phylogenies of the virus species in *Furovirus* implying the independent segregation of the virus subgenomes.

## Introduction

*Japanese soil-borne wheat mosaic virus* (JSBWMV) belongs to the genus *Furovirus*, a set of rigid, rod-shaped bipartite RNA viruses harboring the two positive sense strands RNA1 and RNA2 (Shirako *et al.* 2000). Other known members of the *Furovirus* genus include *Chinese wheat mosaic virus* (CWMV), *Soil-borne wheat mosaic virus* (SBWMV), *Soil-borne cereal mosaic virus* (SBCMV), *Oat golden stripe virus* (OGSV) and *Sorghum chlorotic spot virus* (SrCSV) (Mayo 2005). The type species (SBWMV) is able to colonize small grain cereal species, and can induce significant losses in crop productivity (Cadle-Davidson *et al.* 2006). SBWMV was first identified in 1919 in North America, but has since become widely dispersed across both Europe and east Asia (Bass *et al.* 2006, Clover *et al.* 2001, Hariri and Meyer 2007). The pathogen’s entry into the host plant root is facilitated by an association with the plasmodiophorid root parasite *Polymyxa graminis* (Ohki *et al.* 2017). Infection typically occurs during the fall, after which the virus multiplies *in situ* before being translocated to the host plant’s leaves. Diseased plants develop mosaic patterning on their leaves and their growth is stunted. Crop rotation is ineffective as a control measure because resting *P. graminis* spores can remain viable in the soil for several decades (Brakke and Langenberg 1988), while soil fumigation, although effective, is unacceptable on both economic and ecological grounds. Thus the only practical control measure available is the deployment of resistant cultivars.

Genetic analyses carried out in bread wheat (*Triticum aestivum*) have identified that the presence of the gene *Sbm1* protects the host against SBWMV infection (Brakke and Langenberg 1988, Hao *et al.* 2012, Narasimhamoorthy *et al.* 2006). The same gene, located at the distal end of the long arm of chromosome 5D (Perovic *et al.* 2009), also protects against colonization by the related virus SBCMV. Similarly, *Sbm2*, a gene mapping to the short arm of chromosome 2B (Bayles *et al.* 2007) protects against both SBWMV and SBCMV infection. In durum wheat (*T. turgidum* var. d*urum*), it has been shown that resistance against SBCMV is conferred by a quantitative trait locus (QTL) *QSbm.ubo-2BS* which maps to a chromosomal site similar to that occupied by *Sbm2* (Maccaferri *et al.* 2011, Russo *et al.* 2012). An additional, but less effective, QTL is present on chromosome 2A (Maccaferri *et al.* 2011). Synteny suggests that *Sbm2* and the two durum wheat loci are likely orthologs.

Resistance in barley to the *Bymovirus* pathogen *Barley yellow mosaic virus* is controlled by at least 20 genes, with interactions between them and differing virus strains determining the overall host/pathogen response (Jiang *et al.* 2020). Genes conferring resistance to this virus were cloned by map-based approach (Stein *et al.* 2005, Yang *et al.* 2014). The Netsu *et al.* (2011) serological diagnostic test for SBWMV has been used to search for variation in the response of barley to JSBWMV infection (Aoki *et al.* 2015). As a result, it has been possible to locate a fully dominant gene conferring resistance to this pathogen to the distal end of the short arm of chromosome 2H (Okada *et al.* 2020). However, the identity of genes conferring resistance to JSBWMV, as well as any specificity to particular pathogen strains remains unexplored. The present paper reports the outcome of a QTL analysis based on a mapping population bred from a cross between two related barley cultivars, as well as a determination of the phylogenetic status of JSBWMV.

## Materials and Methods

### Plant materials

The mapping population was developed from a cross between cultivar (cv.) “Tochinoibuki” and cv. “Sukai Golden”, both of which are two-rowed malting barleys bred at the Tochigi Prefectural Agricultural Experimental Station (Takayama *et al.* 2011, Taniguchi *et al.* 2001). The former cultivar was used as the female parent, and the resulting F_1_ hybrid was repeatedly self-pollinated to generate a set of 93 F_6_ and F_7_ recombinant inbred lines (RILs).

### JSBWMV infection

Plants were exposed to JSBWMV infection by raising them in a disease nursery at Yawara (Tsukuba-Mirai, Ibaraki, Japan). Both parental cultivars, their F_1_ hybrid, a sample of F_2_ progeny and the set of F_6_ RILs were sown on 21 October 2015 and the leaves of eight plants per RIL were sampled first on both 16 March 2016 (Rep. #1) and 6 April 2016 (Rep. #2). Both parents and the set of F_7_ RILs were sown on 21 October 2016, with leaf sampling timed for 16 March 2017 (Rep. #3) and 4 April 2017 (Rep. #4).

### ELISA

The ELISA used to quantify viral load in the leaf was based on a polyclonal antibody raised against SBWMV by Netsu *et al.* (2011) from virus prepared from diseased wheat leaves according to Shirako and Brakke (1984). Each sample (5–8 plants per each RIL) was represented by 80 mg leaf tissue, which was homogenized using an MM300 mixer mill (Retsch, Haan, Germany) in 0.8 mL PBST buffer (8 g/L NaCl, 2.9 g/L Na_2_HPO_4_.12H_2_O, 0.2 g/L KH_2_PO_4_, 0.2 g/L KCl, 0.5 mL/L Tween 20). Following centrifugation (9,050 × g, 5 min, 25°C) in an AR015-24 centrifuge (Tomy, Tokyo, Japan), a 0.1 mL aliquot of supernatant was subjected to a double antibody sandwich-ELISA (Clark 2003). After a 30 min incubation at room temperature, a MODEL686 microplate reader (Bio-Rad, Tokyo, Japan) was used to quantify the absorbance of the supernatant at 405 nm. Five to eight plants per each RIL were tested.

### Plant RNA-Seq for marker development

The methods used for growing plants, RNA isolation, cDNA library preparation and RNA sequencing followed those given by Tanaka *et al.* (2019). In brief, RNA was extracted from both the shoot and the roots of seedlings which had developed a shoot of length of ~5 cm. The resulting RNA-Seq library was sequenced using the MiSeq Reagent Kit V3 (2× 300 bp cycles) on a MiSeq NGS system (Illumina, San Diego, CA, USA) according to the MiSeq System User Guide, and fastq files with a read length of 300 bases were obtained from both ends of the fragments. All sequence data have been deposited in the DDBJ/GenBank/ENA BioProject databases, including relevant links to BioProject accession number PRJDB7665.

### Detection of nucleotide variants

Low quality sequence was removed from the RNA-Seq data set, using the option “illuminaclip:adapter.fa:2:30:10 Leading:15 Trailing:15 Slidingwindow:4:15 Minlen:32” provided within the software package Trimmomatic-0.30 (Bolger *et al.* 2014). The resulting processed paired-end reads were mapped onto the cv. “Haruna Nijo” genome assembly (Sato *et al.* 2016) using the option “--min-intron-length 50 --max-intron-length 10000” provided within the Tophat-2.0.10 program (Kim *et al.* 2013). The same procedure was applied to map the reads onto both the cv. “Haruna Nijo” full-length cDNA data set (Matsumoto *et al.* 2011) and the cv. “Morex” reference genome (Mascher *et al.* 2017). Identification of sequence polymorphism and genotyping was performed using the option “-T HaplotypeCaller --num_cpu_threads_per_data_thread 12 --min_base_quality_score 20 --filter_reads_with_N_cigar” provided within the program GenomeAnalysisTK-3.2-2 (McKenna *et al.* 2010). A customized Perl program was used to identify polymorphisms with a homozygous read depth of no less than two.

The set of 121 nt sequences harboring a polymorphism between cv. “Sukai Golden” and cv. “Tochinoibuki” was mapped onto both the cv. “Haruna Nijo” (Tanaka *et al.* 2013) and cv. “Morex” genomic sequence (Mascher *et al.* 2017) using the gmap function, based on 95% identity and 90% coverage (Wu and Watanabe 2005). The physical position of each variable nucleotide was inferred from the cv. “Morex” genome assembly (Mascher *et al.* 2017). The cv. “Haruna Nijo” genome sequence and transcriptome were downloaded from bex-db (Tanaka *et al.* 2013). A subset of the variant nucleotides was chosen to construct Fluidigm genotyping assays suitable for linkage analysis.

### Linkage analysis

The set of RILs (F_6_) was genotyped using a Fluidigm 96.96 Dynamic Array IFC chip (Standard BioTools, Tokyo), following the SNPtype 96x96 v1 protocol (https://fluidigm.my.salesforce.com/sfc/p/#700000009DAw/a/4u0000019kFV/J5w6q0CKYdlTv5ebT5PYx8Ai2T5oGz.ClYtyMxHAWFg). Polymorphic sites were chosen to identify a population of evenly distributed genetic markers. The resulting linkage map was deduced using JoinMap v4.1 software (Van Ooijen 2006), adopting “RIL” as the population model, and including any loci which remained in the heterozygous state. The maximum likelihood mapping algorithm was chosen, and the LOD threshold was set at 4.0. All other settings used the defaults provided within the software package.

### QTL analysis

Five to eight plants per each RIL were analyzed individually by ELISA and the infection rate (%) was used for QTL analysis. Composite interval mapping was performed using the model 6 forward and backward method of the Zmapqtl procedure implemented in Windows QTL Cartographer v2.5 (Basten *et al.* 2000, Wang *et al.* 2005). A genome-wide LOD threshold consistent with a significance level of 0.05 was determined from a set of 1,000 permutations.

### Virus RNA-Seq

Because the viral RNA lacks a poly-A tail, a total RNA-Seq approach was employed for sequencing the JSBWMV present in infected leaves of cv. “Tochinoibuki” grown in the Yawara JSBWMV nursery field during the 2019–2020 season. Total RNA was extracted from leaf tissue using an RNeasy Plant Mini Kit (Qiagen, Hilden, Germany) and its quality checked using a Bioanalyzer device (Agilent Technologies, Santa Clara, CA, USA). Only RNA samples displaying a RIN value >7 were used for library construction. The libraries were assembled using a TruSeq Stranded Total RNA Library Prep Kit with RiboZero (Illumina) and sequenced using the NovaSeq 6000 NGS system (Illumina). To remove adaptor sequence, the fastq data generated were processed by imposing the Trimmomatic-0.33 program (Bolger *et al.* 2014), applying the option “Illuminaclip:adapters.fa:2:30:10 Leading:20 Trailing:20 Slidingwindow:4:15 Minlen:36”. Paired-end reads were mapped onto the JSBWMV reference sequence (accession LC481573 for RNA1 and LC481574 for RNA2) using the bwa mem aligner program, using default parameters (Li and Durbin 2010). The resulting SAM files were converted to BAM files using the samtools v1.11 program (Li *et al.* 2009). Variant calling was achieved with the aid of the bcftools mpileup program (Li 2011) using the option “-d 1000000 –annotate FORMAT/AD”. Consensus bases were called from allele depth of field of vcf file using a customized perl script.

### Phylogenetic analysis

Multiple alignments of both the RNA nucleotide and the predicted amino acid sequences were performed using the ClustalW algorithm (Larkin *et al.* 2007). Phylogenetic trees were constructed using the maximum likelihood method implemented in MEGA11 software (Tamura *et al.* 2021) according to the general time reversible (nucleotides) or the Jones-Taylor-Thornton (polypeptides) models. Branch support was calculated by 1,000 bootstrap replicates. A discrete Gamma distribution was used to model evolutionary rate differences among sites. Where the alignment implied the existence of an indel, the indel sequence was eliminated using the ‘complete deletion’ option.

## Results

### ELISA-based quantification of JSBWMV titer in barley leaves

**Fig. 1.**
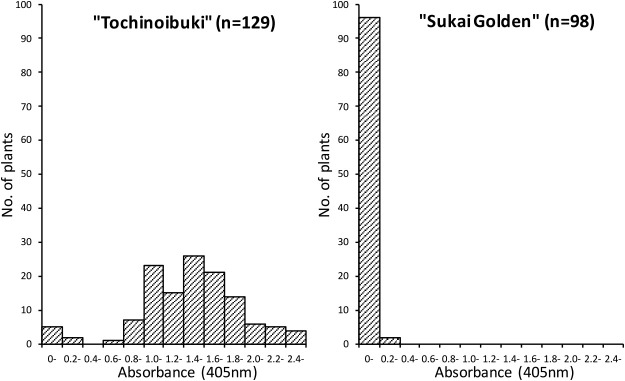
ELISA-derived absorbances of single plants of both of the mapping population parents. Values produced by cv. “Tochinoibuki” plants mostly exceeded 0.6, whereas those produced by cv. “Sukai Golden” plants were concentrated in the range 0–0.4. Plants producing an absorbance value of <0.2 were classified as JSBWMV uninfected and those >1.0 as infected. In order to reduce noise, plants recording a value in the range 0.2–1.0 were excluded from the analysis.

A representative outcome of the ELISA-based quantification of the JSBWMV titer in the leaf is shown in Fig. 1. Samples generating an absorbance value <0.2 were categorized as uninfected and those >1.0 as infected. Samples showing values in the range 0.2–1.0 were excluded from the analysis in order to reduce noise. A preponderance (87–100%) of cv. “Tochinoibuki” plants harbored the virus, demonstrating that the disease pressure in the nursery was high; no virus was detected in any cv. “Sukai Golden” plants (Table 1). Both the cv. “Tochinoibuki” × cv. “Sukai Golden” and the cv. “Sukai Golden” × cv. “Tochinoibuki” F_1_ hybrids were free of virus, showing that the gene(s) responsible for the resistance of cv. “Sukai Golden” were dominant and were nuclear rather than cytoplasmic. Of the 284 cv. “Tochinoibuki” × cv. “Sukai Golden” F_2_ progeny assayed in two independent experiments, 18 proved to be infected and 266 uninfected, consistent with the 1:15 segregation ratio resulting from the presence in cv. “Sukai Golden” of two independent, dominant genes (χ^2^ = 0.0037, df = 1, P > 0.95).

### Development of markers by RNA-Seq polymorphisms

A set of 7,508,510 paired reads was recovered from cv. “Tochinoibuki” shoot and root tissue and 4,169,883 from cv. “Sukai Golden” shoot and root tissue (Supplemental Table 1). After trimming, the numbers of reads were reduced to, respectively, 7,287,654 and 4,067,120; of these, respectively, 4,799,868 (65.9%) and 2,728,965 (67.1%) were successfully mapped onto the cv. “Haruna Nijo” genome sequence; somewhat lower proportions (56.7% and 48.5%) matched sequences present in the cv. “Morex” genome sequence. The RNA-Seq data were finally assembled into a set of 36,303 presumptive genes, of which 26,226 were mappable to a specific chromosomal site in the cv. “Morex” genome (Supplemental Table 2). The number of sites which were polymorphic between cv. “Tochinoibuki” and cv. “Sukai Golden” was 7,475, compared to 9,843 for cv. “Sukai Golden”/cv. “Haruna Nijo” and 12,250 for cv. “Tochinoibuki”/cv. “Haruna Nijo”. When the genomic location of 3,737 of the cv. “Tochinoibuki” and cv. “Sukai Golden” sites was deduced by reference to the cv. “Morex” genome sequence, it was apparent that they were distributed across all seven chromosomes, but with a markedly lower density along chromosomes 5H and 6H. Inspection of the pedigrees of cv. “Tochinoibuki” and cv. “Sukai Golden” implied that 25% of their genomes were inherited from “Tochikei 216” and 12.5% from cv. “Haruna Nijo” (Takayama *et al.* 2011, Taniguchi *et al.* 2001). Those polymorphisms were used to design the Fluidigm chip.

### Linkage mapping

The sequences of each of the allele-specific, locus-specific and specific target amplification primers are given in Supplemental Table 3. SNP genotyping at 192 sites using the Fluidigm 96.96 IFC chip platform realized 166 polymorphisms, which segregated across the 93 F_6_ RILs (Supplemental Table 4). The resulting linkage analysis ordered the informative 132 markers into 13 linkage groups (LGs), ranging in length from 0.6 to 118.6 cM (Supplemental Fig. 1). Gaps along chromosomes 2H, 3H, 5H and 7H were responsible for the number of LGs being greater than the haploid number of seven. The chromosome 6H map was particularly short (18 cM). Reducing the LOD threshold to 3.0 did not reduce the number of LGs identified. The entire map comprised just 496 cM (Supplemental Table 2), a length which is under half that determined from other biparental crosses (Graner *et al.* 2011). The presence of gaps and the foreshortening of the map are assumed to reflect the relatively close relationship between the two parental cultivars. Among 166 markers, 34 were not shown in the linkage map (Supplemental Fig. 1), either because in the case of multiple markers mapping to same locus, only one was retained (27 markers), or the program assigned them to an unexpected chromosome (three markers), or the program was unable to place the marker within an LG (four markers) (Supplemental Table 4).

### QTL underlying JSBWMV resistance expressed by cv. “Sukai Golden”

**Fig. 2.**
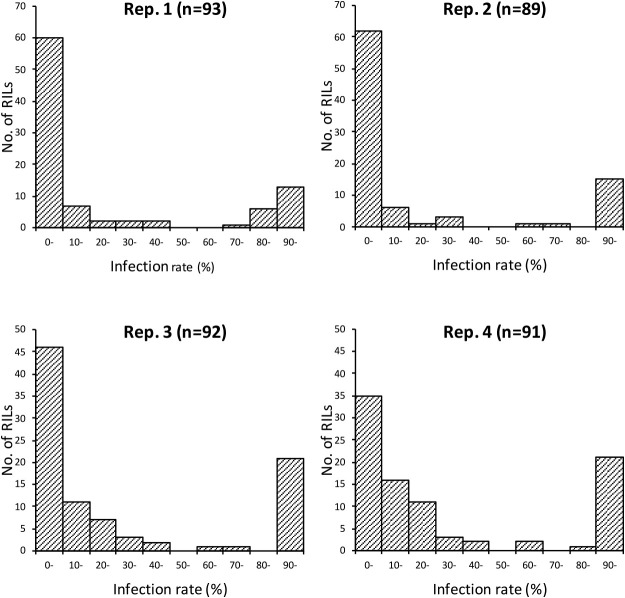
Frequency distribution of JSBWMV reaction in the RILs bred from the cross between cv. “Tochinoibuki” and cv. “Sukai Golden”.

**Fig. 3.**
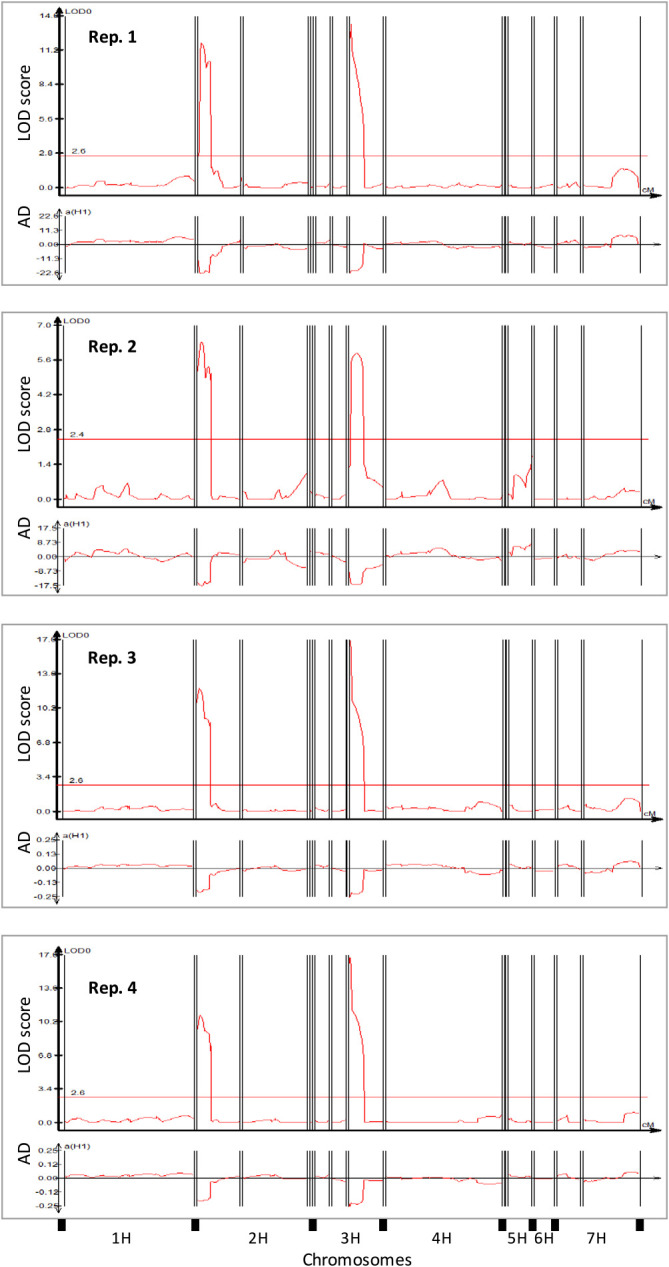
Genetic analysis of JSBWMV resistance. Each chromosome represented on the *x* axis is represented with its short arm on the left. The *y* axes record either the LOD score (upper trace) or the additive effect of a QTL allele (lower trace). A negative additive effect in 2H and 3H indicates that the cv. “Sukai Golden” allele promotes resistance. Vertical black lines are inter- and intra-chromosomal gaps of linkage.

The reaction of all individual plants across the RILs was scored binomially as uninfected or infected according to the viral titer assayed by the ELISA (Supplemental Fig. 2). The distribution of infection rate among the RILs was clearly bimodal (Fig. 2). A QTL analysis predicted the presence of two major loci as responsible for the bulk of the phenotypic variation: one of these mapped close to the terminus of chromosome arm 2HS, and the other within chromosome 3HL (Fig. 3). Across the four replicates, the former locus was associated with a LOD score of 6.3–12.2, with the peak of the QTL trace indicating that the gene underlying the QTL was located ~3 cM from the 2HS telomere (Table 2). The allele presented in cv. “Sukai Golden” explained 21–32% of the phenotypic variation measured in the population. The chromosome 3H QTL was also reproducibly detected across all four replicates, was associated with a LOD score of 5.9–16.9 and the peak of the trace implied a location of the underlying gene between 0–6.7 cM (position) along LG7, defined by the markers FB0245 and FB0246, which sets its position between 0 cM and 13.6 cM of the LG7 in chromosome 3HL. The relative contribution of the cv. “Sukai Golden” allele to the population’s phenotypic variation was 20–37%.

### Mapping of JSBWMV resistance genes as monogenic factors

The set of RILs could be divided into two sub-populations on the basis of zygosity at each of the two QTL regions in turn. The ‘Sub-3H.TI’ sub-population members comprised homozygotes for the cv. “Tochinoibuki” allele at all loci in the interval bounded by FB0245 and FB0246, while the ‘Sub-3H.SG’ sub-population members were all homozygous for the cv. “Sukai Golden” alleles in this interval (Table 3). All ‘Sub-3H.SG’ members were JSBWMV resistant, while across all four replicates, there was a 1:1 segregation for resistance/susceptibility among the ‘Sub-3H.TI’ members (Supplemental Fig. 3). For the equivalent two sub-populations constructed on the basis of zygosity at the chromosome 2H loci bounded by FB0209 and FB0214 (‘Sub-2H.TI’ and ‘Sub-2H.SG’), half of the ‘Sub-2H.TI’ members proved to be JSBWMV resistant and half susceptible, while all of the ‘Sub-2H.SG’ members were resistant. This analysis implied the presence of two independent genes conferring resistance to JSBWMV.

**Fig. 4.**
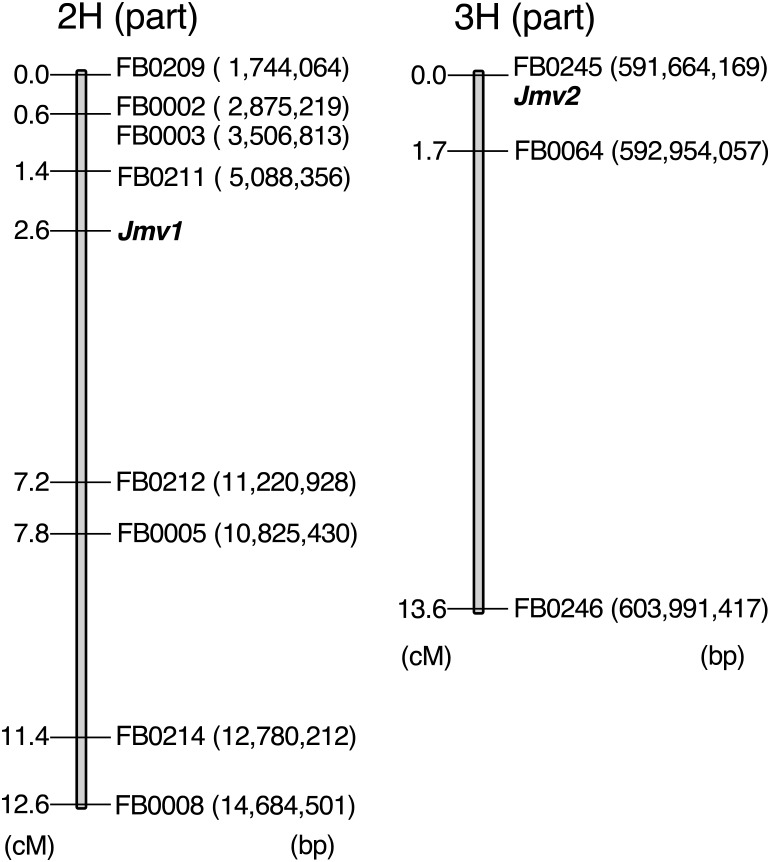
Genetic maps of parts of chromosomes 2H and 3H harboring the genes underlying the JSBWMV resistance expressed by cv. “Sukai Golden”. The physical location of markers was derived from Mascher *et al.* (2021).

Linkage mapping using the appropriate genetic markers and ELISA scores in segregating RILs (Supplemental Table 5) placed the 2HS resistance locus (here assigned the symbol *Jmv1*) within a 5.8 cM/6.2 Mbp interval flanked by FB0211 (physical position 5 Mb) and FB0212 (11.2 Mb) (Fig. 4). The physical location of markers was derived from Mascher *et al.* (2021). Meanwhile, the 3HL locus (*Jmv2*) and FB0245 (591.6 Mb) didn’t recombine (Supplemental Table 6), so no conclusion could be drawn as to whether it lies distal or proximal to the marker. Among the full set of RILs, there was a 3:1 segregation ratio between resistant and susceptible (Table 3), as expected for two non-linked genes (in this case *Jmv1* and *Jmv2*).

**Fig. 5.**
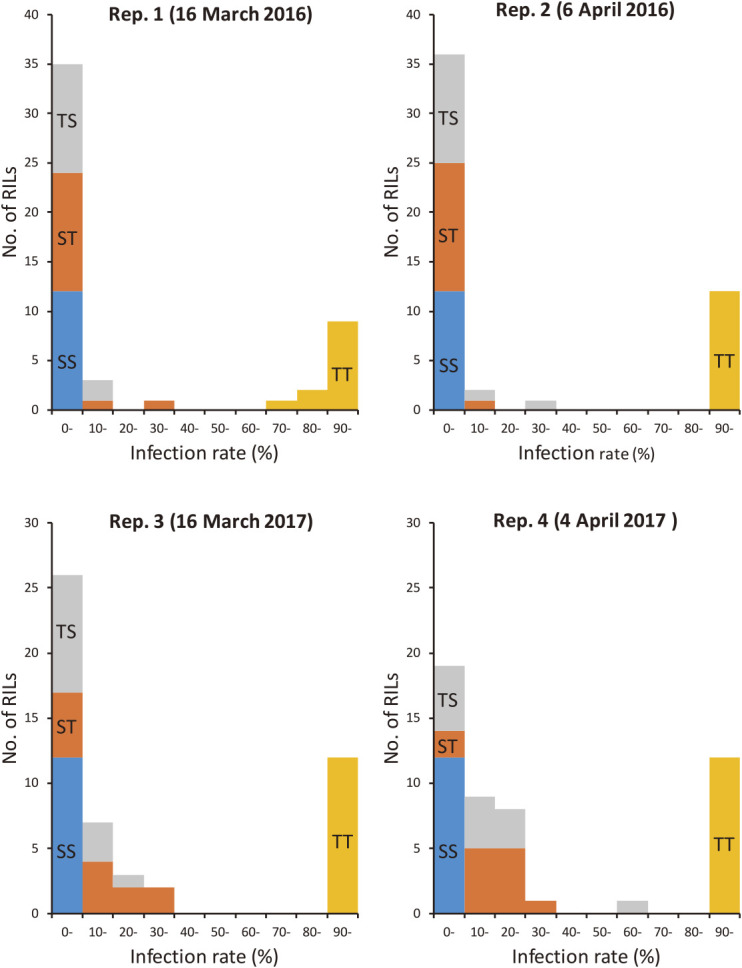
Distribution of JSBWMV infection rate in RILs bred from the cross between cv. “Tochinoibuki” and cv. “Sukai Golden”. RILs homozygous at each gene were sub-populated (n = 51), where genotype of *Jmv1* was inferred by FB0209 and FB0008 and genotype of *Jmv2* by FB0245 and FB0246. SS - “Sukai Golden” genotype for *Jmv1* and *Jmv2*; TT - “Tochinoibuki” genotype for *Jmv1* and *Jmv2*; ST - “Sukai Golden” genotype for *Jmv1* and “Tochinoibuki” genotype for *Jmv2*; TS - “Tochinoibuki” genotype for *Jmv1* and “Sukai Golden” genotype for *Jmv2*.

Both *Jmv1* and *Jmv2* alone provided a partial level of field susceptibility, while RILs carrying both genes in the homozygous state universally escaped infection in 2016 (Fig. 5). In 2017 RILs that carried either *Jmv1* or *Jmv2* alone showed an increased infection rate (<40%), while RILs carrying both genes were essentially immune. The result implied that the two dominant genes complementarily encode perfect resistance to JSBWMV.

### Virus resequencing

Of the paired reads generated by the total RNA-Seq, 1.0% matched sequences represented in the RNA1 reference sequence, as did 1.7% to the RNA2 reference sequence. There was no between duplicate sequence variation between reads matching the RNA1 sequence, but there was one instance of a single nucleotide polymorphism (T6304A) uncovered between the reference RNA1 sequence LC481573 and sequence LC642564: this variant is predicted to generate a L2102M alteration in the virus’ 37 kDa movement protein. Sequence LC642565 featured heterogeneity at two sites (T346C and A1534G) with respect to the type RNA2 sequence LC481574, with, respectively, the T and A nucleotide matching the reference sequence. However, neither variant is predicted to generate any change at the polypeptide level. The first open reading frame of RNA1 encodes a 152 kDa protein including a putative methyltransferase domain and a helicase domain; its read-through at the leaky opal stop codon results in the translation of an additional 59 kDa RNA polymerase. The second open reading frame encodes a 37 kDa movement protein. The first open reading frame of RNA2 encodes a 19 kDa capsid protein and a 64 kDa read-through protein, while its second open reading frame encodes a 19 kDa cysteine-rich protein. These analyses replicated the results of Shirako *et al.* (2000) and supported that this virus belonged to JSBWMV”.

### JSBWMV phylogeny

**Fig. 6.**
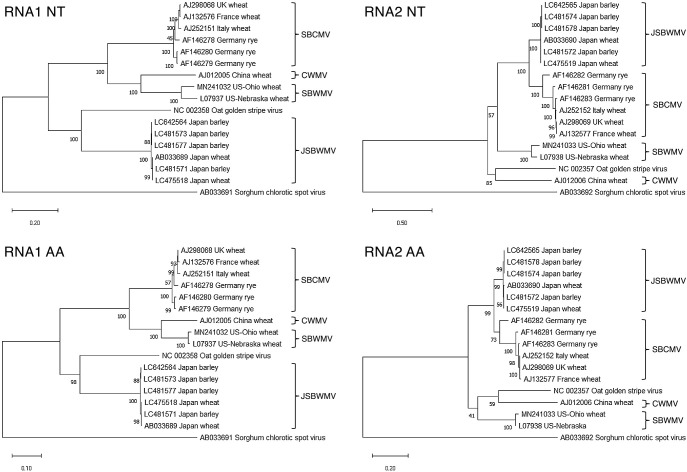
Phylogenetic analysis of the genomic RNA sequences and deduced polypeptide sequences in a set of *Furovirus* soil-borne viruses: *Japanese soil-borne wheat mosaic virus* (JSBWMV), *Soil-borne cereal mosaic virus* (SBCMV), *Chinese wheat mosaic virus* (CWMV), *Soil-borne wheat mosaic virus* US-isolate (SBWMV), *Oat golden stripe virus* (OGSV) and *Sorghum chlorotic spot virus* (SrCSV). LC642564 and LC642565 are the sequences acquired in the present study. Virus RNA was purified from the leaves of susceptible cv. “Tochinoibuki” plants grown in a JSBWMV-infested field in Yawara, Ibaraki. Bootstrap support percentages for each branch were obtained from 1,000 replicates. NT - nucleotide sequence, AA - amino acid sequence of concatenated proteins.

A phylogenetic analysis based on six known *Furovirus* species (JSBWMV, SBCMV, CWMV, SBWMV, OGSV and SrCSV) is shown in Fig. 6. The clade harboring JSBWMV RNA1 included the type RNA1 sequence LC642564 from this study. The OGSV RNA1 sequence and its JSBWMV equivalent formed a clade with 100% bootstrap support, while the SBCMV, CWMV and SBWMV RNA1s formed a distinct clade, leaving SrCSV as an outgroup. An identical phylogeny was deduced using polypeptide sequences predicted from the RNA1 sequences (three concatenated proteins). The equivalent analysis of the RNA2 sequences clustered the sequence LC642565 from this study within the same clade which harbored JSBWMV, and showed that the JSBWMV sequence was closely related to that harbored by SBCMV with 100% bootstrap support. The phylogenetic relationships between the SBWMV, CWMV and OGSV RNA2’s was not strongly supported by bootstrap analysis.

## Discussion

### The genetic basis of JSBWMV resistance in cv. “Sukai Golden”

Most of the genes conferring resistance in barley to *Barley yellow mosaic virus* act as recessive alleles (Jiang *et al.* 2020). Members of the *rym4/rym5/rym6/rym10* cluster each encode an eIF4E variant which promotes resistance (Stein *et al.* 2005), while *rym1* and *rym11* both encode a PDIL5-1 variant (Yang *et al.* 2014). The only three genes known to act dominantly are *Rym14^Hb^* and *Rym16^Hb^*, both of which have been introgressed into cultivated barley from its wild relative *H. bulbosum* (Ruge *et al.* 2003, Ruge-Wehling *et al.* 2006), and *Rym17*, identified in a cultivar bred in Pakistan (Kai *et al.* 2012). In contrast, genes responsible for the resistance of wheat to *Wheat yellow mosaic virus* all act dominantly (Suzuki *et al.* 2015). Likewise, resistance to JSBWMV in barley appears to be dominant, as shown both by Okada *et al.* (2020) and by the behavior of *Jmv2* and *Jmv1*. Four pieces of direct evidence support the notion that *Jmv1* is identical to the gene identified by Okada *et al.* (2020): firstly, the same genomic location is involved; secondly, both resistance genes were completely dominant over the recessive alleles; thirdly, the source of inoculum used was the same; and fourthly cv. “Haruna Nijo” is one of the ancestors of cv. “Sukai Golden”. The proposition is supported by the observation that in the RIL population, the two resistant genes inherited from cv. “Sukai Golden” explained around a half (41–63%) of the variation for JSBWMV infection rate, while according to Okada *et al.* (2020), the gene harbored by cv. “Haruna Nijo” only explained 33–40% variation seen in a mapping population.

Given that they both act as dominant genes, *Jmv1* and *Jmv2* are potentially functionally similar to one another. There are plenty of examples of genes which act in a dominant manner among the members of the diverse families of plant genes conferring resistance against a variety of viruses (Ronde *et al.* 2014). A similar interaction has been noted for the two wheat genes *Ym1* and *Ym2* which encode resistance to *Wheat yellow mosaic virus* (Suzuki *et al.* 2022). Carriers of just one of either of these two genes are only partially resistant, while lines carrying both are essentially immune (Suzuki *et al.* 2015). Combined gene effect of *Jmv1* and *Jmv2* are similar with the two wheat resistance genes.

### Potential orthology of *Jmv1* and *Jmv2* with wheat genes determining resistance to *Furovirus* species pathogens

Resistance in durum wheat to the *Furovirus* pathogen SBCMV is conferred by the two homeoloci *Qsbm.ubo-2AS* and -*2BS* (Maccaferri *et al.* 2011, 2012). Since their map location is syntenic to that of *Jmv1*, it is likely that these three genes are orthologs of one another. Genetic analysis in bread wheat has identified a number of minor QTL underlying variation in SBCMV resistance, located variously on chromosome arms 2AL, 3AL, 3BL, 5AL and 6BS. The two present on the group 3 homeologs, which are homeologous with barley chromosome 3H, thus may represent potential orthologs of *Jmv2*. Mapping carried out in barley itself has identified a number of putative JSBWMV resistance QTL located on chromosome arms 2HL, 3HL, 5HL and 6HS (Okada *et al.* 2020), but their effect (if any) was too small to be detected in the present study.

### Choosing closely related lines as mapping population parents risks a low global level of marker polymorphism, but only in non-target regions of the genome

The relative shortness of the linkage map derived from the mapping population and the presence of within chromosome gaps was a consequence of a level of relatedness between the two parental cultivars cv. “Tochinoibuki” and cv. “Sukai Golden”, both of which were developed at the same experimental station over the course of the last 20 years (Takayama *et al.* 2011, Taniguchi *et al.* 2001). Any genomic region inherited intact from their common ancestor by both mapping population parents will necessarily be monomorphic with respect to any marker or trait locus (such as JSBWMV resistance) located within it. Nevertheless, the strategy of applying RNA-Seq, followed by a mapping of the resulting reads onto the cv. “Morex” reference genome, as suggested by Tanaka *et al.* (2019), was successful in developing tags for both of the JSBWMV resistance genes present in cv. “Sukai Golden”. The future use of closely related parental genotypes should be facilitated by the recent acquisition of chromosome-scale sequence assemblies of 20 barley lines (Jayakodi *et al.* 2020).

### JSBWMV strain

The phylogenetic analysis based on the two viral RNA sequences was able to identify a number of differences between European (SBCMV), Japanese (JSBWMV), Chinese (CWMV) and US (SBWMV) species. The joint presence of both *Jmv1* and *Jmv2* protects field-grown barley from infection by the local JSBWMV strain. Intriguingly, there was a difference between the RNA1- and RNA2-based phylogenies of the strain. Thus, for example, based on the RNA1 sequence, it appeared to most closely related to OGSV, whereas based on the RNA2 sequence, the closest related sequence was harbored by SBCMV. The reason for this inconsistency remains obscure, although a likely cause lies in the independent segregation of the virus subgenomes, which is advantageous to the pathogen’s adaptation to hosts and ecological contexts (Lucía-Sanz and Manrubia 2017). The joint presence of both *Jmv1* and *Jmv2* was shown here to protect field-grown barley from JSBWMV infection, at least by the local strain of the virus. The ability of either (or both) of these resistance genes to protect barley against infection by other strains of JSBWMV and even perhaps by other *Furovirus* species remains to be established.

## Author Contribution Statement

K.O., T.T., T.Ko, K.N. and T.Ka conceived the experiments, T.Ka developed the germplasm, K.O., K.N. and T.Ka carried out the ELISA experiments, K.S. performed the RNA-Seq analysis, T.T. was responsible for the detection of nucleotide variation and S.F. for genotyping, K.O. and T.O. carried out the QTL mapping, Y.O., K.M. and T.O. performed RNA-Seq on the virus, and K.O., T.T., S.F., Y.O., T.O. and T.Ko wrote the paper.

## Supplementary Material

Supplemental Figures

Supplemental Table 1

Supplemental Table 2

Supplemental Table 3

Supplemental Table 4

Supplemental Table 5

Supplemental Table 6

## Figures and Tables

**Table 1.** The reaction to JSBWMV infection of the mapping population’s parents and early generation progeny

Plants	Rep. 1 (March 2016)				Rep. 2 (April 2016)				Rep. 3 (March 2017)				Rep. 4 (April 2017)		
	*n*	*inf*	%		*n*	*inf*	%		*n*	*inf*	%		*n*	*inf*	%
Tochinoibuki	24	21	88		22	20	91		38	38	100		40	40	100
Sukai Golden	8	0	0		8	0	0		40	0	0		40	0	0
F_1_ TI × SG	22	0	0		23	0	0		0	*n.a.*	*n.a.*		0	*n.a.*	*n.a.*
F_1_ SG × TI	23	0	0		23	0	0		0	*n.a.*	*n.a.*		0	*n.a.*	*n.a.*
F_2_ TI × SG	70	1	1		66	5	8		0	*n.a.*	*n.a.*		0	*n.a.*	*n.a.*
F_2_ SG × TI	75	9	12		73	3	4		0	*n.a.*	*n.a.*		0	*n.a.*	*n.a.*

*n* - number of plants tested, *inf* - number of plants infected, % - infection rate, TI - cv. “Tochinoibuki”, SG - cv. “Sukai Golden”. *n.a.* - not applicable.

**Table 2.** QTL analysis targeting the genetic control of resistance to JSBWMV in cv. “Sukai Golden”

Replication	Chr.	LG	Position (cM)	Closest	Interval markers (position, cM)	LOD	R^2^ (%)	AD (%)	Resistant allele
Rep. 1	2H	LG02	2.41	FB0211	FB0209–FB0214 (0 to 11.4)	11.7	31.6	22.6	Sukai Golden
do.	3H	LG07	1.01	FB0064	FB0245–FB0246 (0 to 13.6)	13.3	31.6	22.6	Sukai Golden
Rep. 2	2H	LG02	3.41	FB0211	FB0209–FB0008 (0 to 12.6)	6.3	21.4	17.4	Sukai Golden
do.	3H	LG07	6.71	FB0064	FB0245–FB0246 (0 to 13.6)	5.9	19.8	16.8	Sukai Golden
Rep. 3	2H	LG02	2.41	FB0211	FB0209–FB0008 (0 to 12.6)	12.2	25.2	20.7	Sukai Golden
do.	3H	LG07	0.01	FB0245	FB0245–FB0246 (0 to 13.6)	16.9	37.1	25.3	Sukai Golden
Rep. 4	2H	LG02	2.41	FB0211	FB0209–FB0008 (0 to 12.6)	10.9	24.1	20.4	Sukai Golden
do.	3H	LG07	0.01	FB0245	FB0245–FB0246 (0 to 13.6)	16.7	36.9	24.8	Sukai Golden

R^2^ - Proprtion of observed variation in resistance explained; AD - additive effect of the allele for infection rate.

**Table 3.** Segregation for resistance/susceptibility among the full RIL population and selected sub-populations

Population*^a^*	Genotype at QTL in		Resistant	Observed*^b^*			Expected*^c^*		χ^2^	d.f.	P
	chr. 2H	chr. 3H		Replication	Susceptible		Resistant	Susceptible			
Sub-3H.TI	segregate	“Tochinoibuki”	Rep. 1	22	17		19.5	19.5	0.64	1	0.42
			Rep. 2	22	16		19.0	19.0	0.95	1	0.33
			Rep. 3	19	18		18.5	18.5	0.03	1	0.87
			Rep. 4	19	18		18.5	18.5	0.03	1	0.87
Sub-3H.SG	segregate	“Sukai Golden”	Rep. 1	34	0		17.0	17.0	34.00	1	0.00
			Rep. 2	34	0		17.0	17.0	34.00	1	0.00
			Rep. 3	34	0		17.0	17.0	34.00	1	0.00
			Rep. 4	38	0		19.0	19.0	38.00	1	0.00
Sub-2H.TI	“Tochinoibuki”	segregate	Rep. 1	18	16		17.0	17.0	0.12	1	0.73
			Rep. 2	18	16		17.0	17.0	0.12	1	0.73
			Rep. 3	17	17		17.0	17.0	0.00	1	1.00
			Rep. 4	16	17		16.5	16.5	0.03	1	0.86
Sub-2H.SG	“Sukai Golden”	segregate	Rep. 1	35	0		17.5	17.5	35.00	1	0.00
			Rep. 2	35	0		17.5	17.5	35.00	1	0.00
			Rep. 3	34	0		17.0	17.0	34.00	1	0.00
			Rep. 4	34	0		17.0	17.0	34.00	1	0.00
Full RIL	segregate	segregate	Rep. 1	73	20		69.8	23.3	0.61	1	0.44
			Rep. 2	72	16		66.0	22.0	2.18	1	0.14
			Rep. 3	69	22		68.3	22.8	0.03	1	0.86
			Rep. 4	67	22		66.8	22.3	0.00	1	0.95

*^a^* The two Sub-3H sub-populations comprise lines homozygous for the allele inherited from one or the other mapping population parent at markers in the genetic interval flanked by FB0245 and FB0246; the two Sub-2H sub-populations comprise lines homozygous for the allele inherited from one or the other mapping population parent at markers in the genetic interval flanked by FB0209 and FB0008. *^b^* Sum of two sub-populations were less than that of full population because heterozygous and recombinant lines in the interval were excluded. *^c^* A χ^2^ test was used to test for a fit with a 1:1 segregation in each sub-population and with a 3:1 segregation in the full RIL population.
