# Supplementary material for: Two dominant genes in barley (Hordeum vulgare L.) complementarily encode perfect resistance to Japanese soil-borne wheat mosaic virus
Source: Breed Sci. 2022 Dec 13;72(5):372–82. doi: 10.1270/jsbbs.22046 (PMC9895801; doi:10.1270/jsbbs.22046)
Supplement: Supplementary file 1 — Supplemental Figures [file 72_372_s1.pdf]

Okada et al./Breeding Science
doi: 10.1270/jsbbs.22046

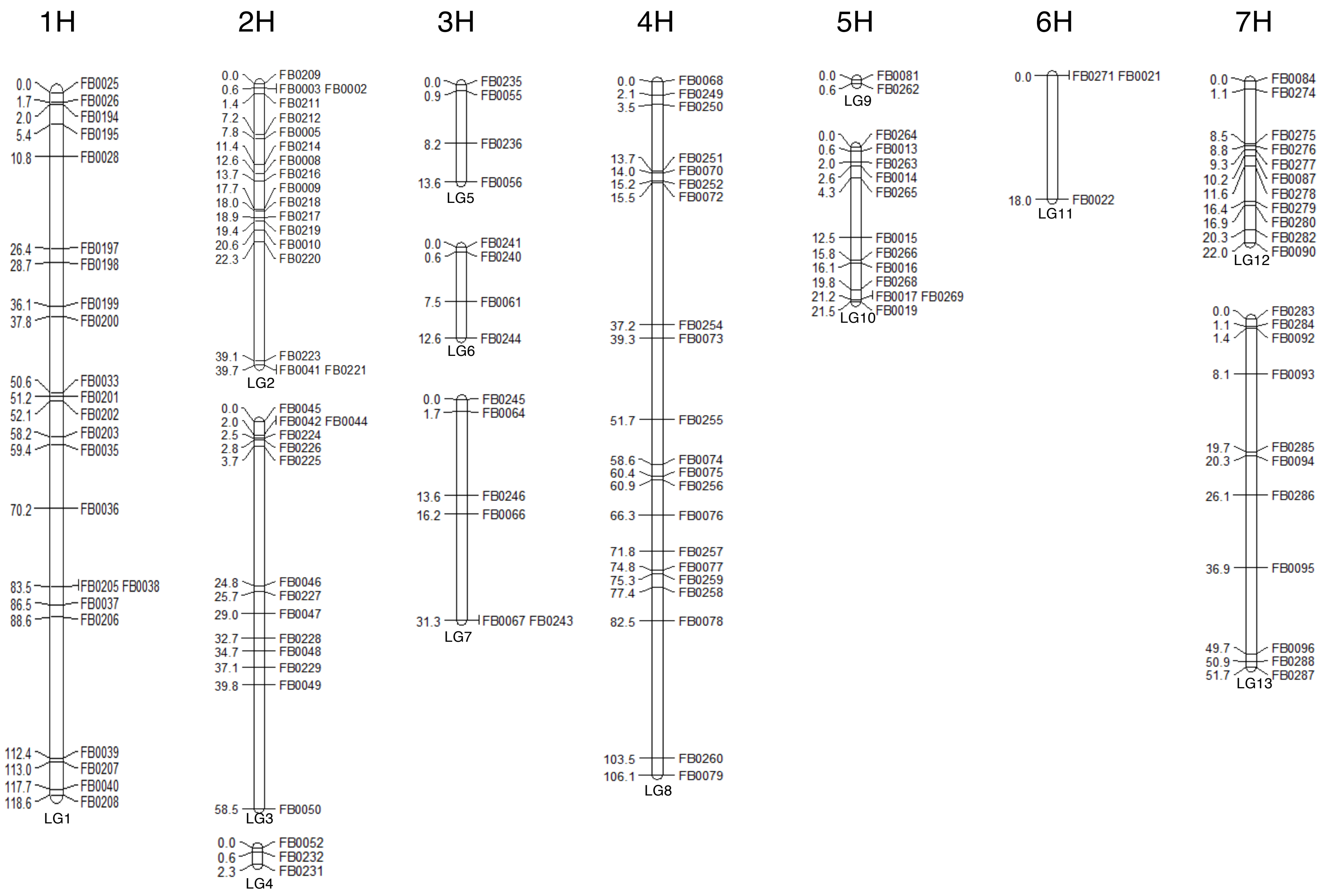

Supplemental Fig. 1. Linkage map derived from segregation among the RILs bred from the cross between cv. "Tochinoibuki" and cv. "Sukai Golden." The markers were developed from the output of an RNA-Seq analysis and were assayed using the Fluidigm platform.

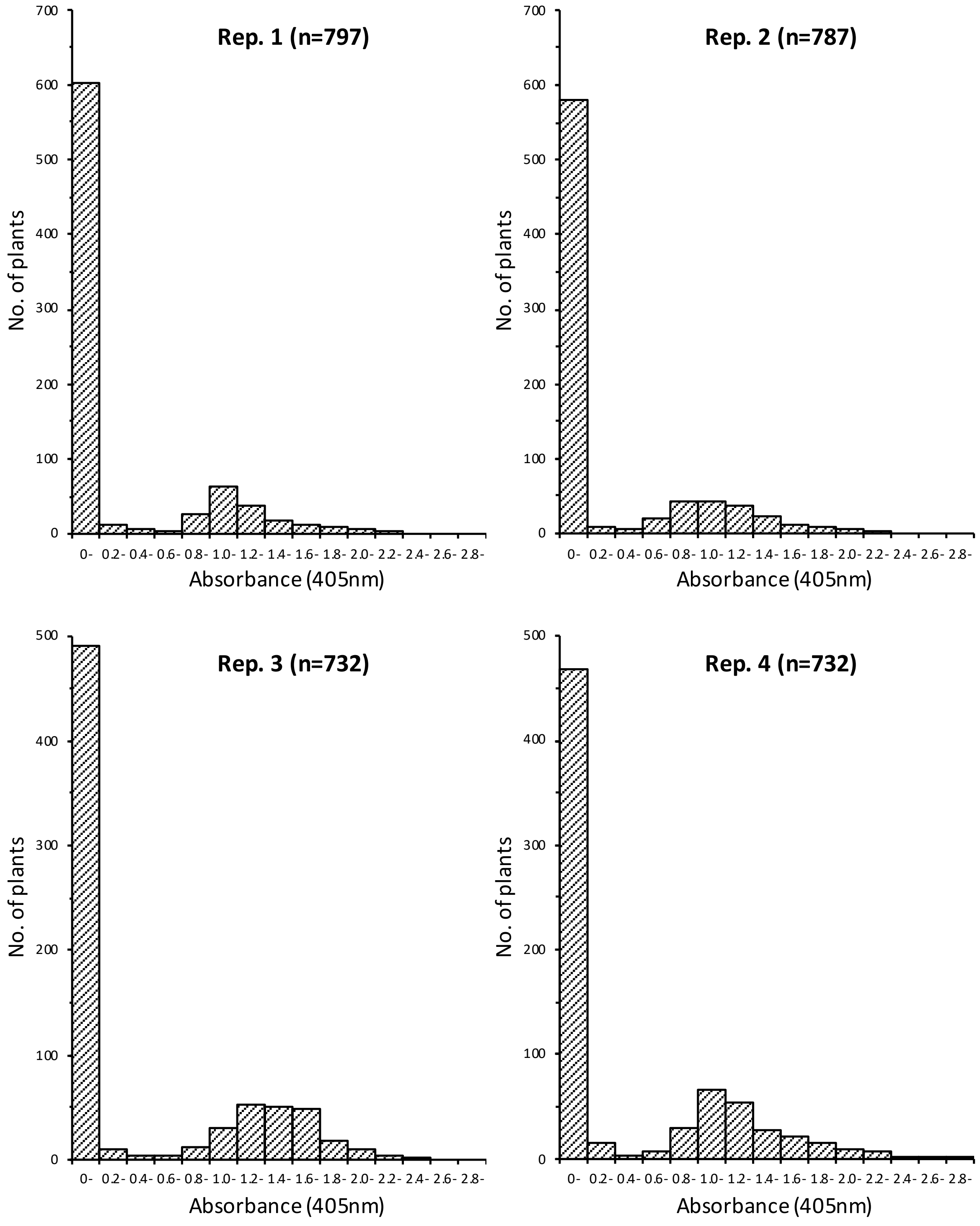

Supplemental Fig. 2. The bimodal distribution of ELISA absorbances produced by the RILs bred from the cross between cv. "Tochinoibuki" and cv. "Sukai Golden". Plants producing an absorbance value of <0.2 were classified as JSBWMV uninfected and those a value >1.0 (except for >0.8 in Rep. 4) as infected. In order to reduce noise, plants recording a value in the range 0.2-1.0 (except for 0.2-0.8 in Rep. 4) were excluded from the count of infection rate. In the first replicate, 602 individuals were scored as JSBWMV resistant, 147 as susceptible and 48 as unclassified; the equivalent numbers for the other three replicates were, respectively, 580, 133 and 74; 491, 214 and 27; and 469, 235 and 28.

Sub-3H.TI (3H QTL= Homo cv. "Tochinoibuki")

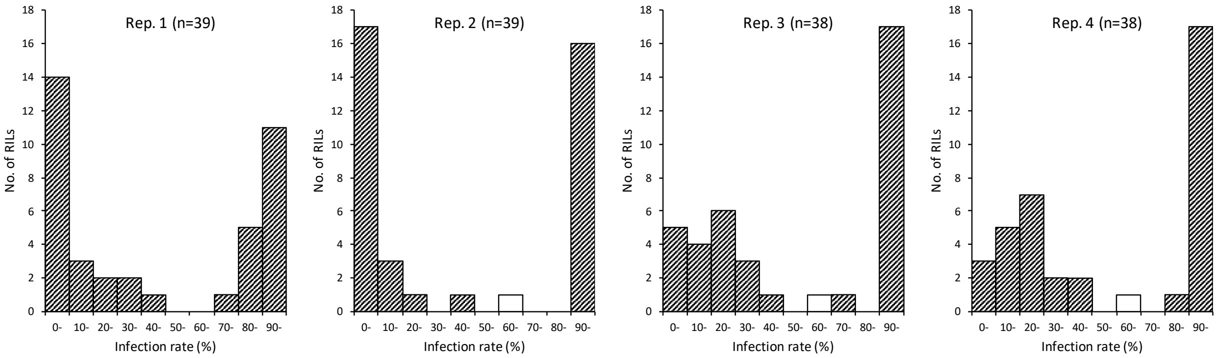

Sub-3H.SG (3H QTL= Homo cv. "Sukai Golden")

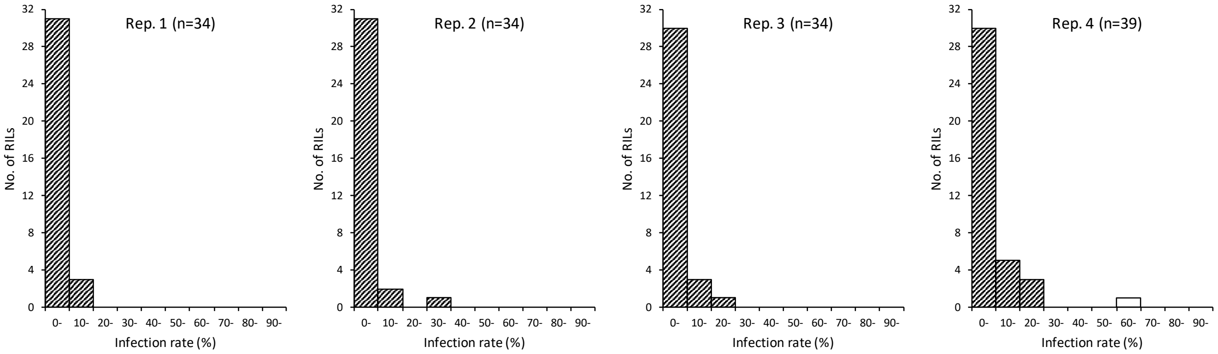

Sub-2H.TI (2H QTL= Homo cv. "Tochinoibuki")

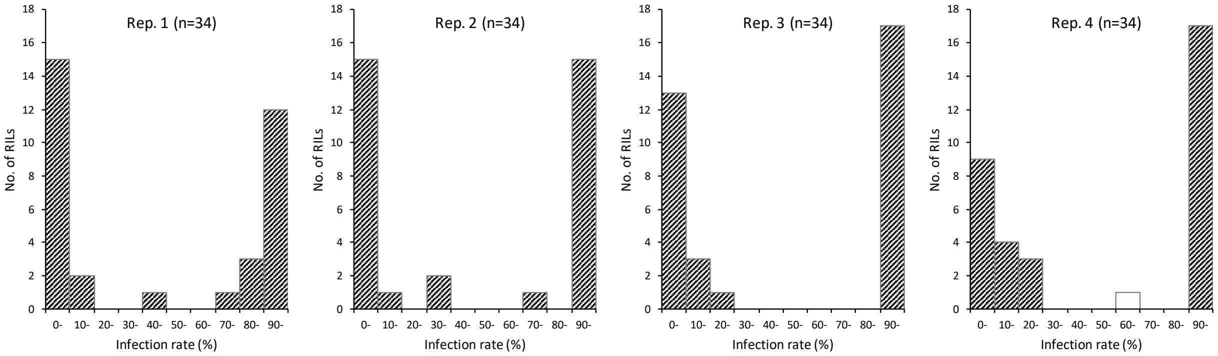

Sub-2H.SG (2H QTL= Homo cv. "Sukai Golden")

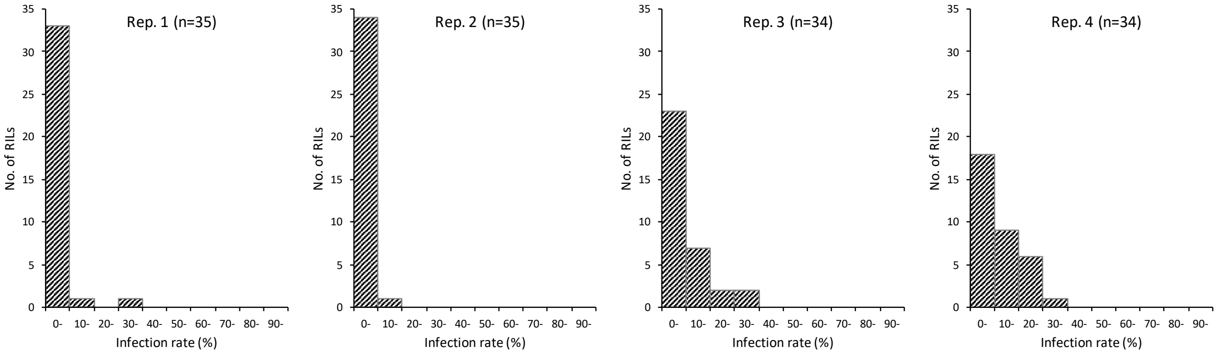

Supplemental Fig. 3. Distribution of JSBWMV infection rate in sub-populations from the RILs bred from the cross between cv. "Tochinoibuki" and cv. "Sukai Golden". RILs showing infection rate ≤ 50% were classified as resistance and those a rate ≥70% as suscepible. In order to reduce noise, RILS recording an infection rate in the range 50-70% (open bars) were excluded from the linkage analysis.
